# L'ostéome ostéoïde du toit de l'acétabulum: une localisation exceptionnelle

**DOI:** 10.11604/pamj.2015.21.134.7088

**Published:** 2015-06-17

**Authors:** Fatima Zahra Rhouni, Youness Sasbou

**Affiliations:** 1 Service d'Imagerie Médicale, Hôpital Militaire d'Instruction Mohammed V, Rabat, Maroc; 2 Service de Traumatologie-Orthopédie, Hôpital Militaire d'Instruction Mohammed V, Rabat, Maroc

**Keywords:** Ostéome, ostoïde, acétabulum, osteoma, osteoid, acetabulum

## Image en medicine

L'ostéome ostéoïde représente 10 % environ des tumeurs osseuses bénignes. Il se localise de façon préférentielle au niveau du fémur (40%). l'atteinte acétabulaire est rare (moins de 1 % selon Campanacci) et celle du toit de l'acétabulum est exceptionnelle. L'ostéome ostéoïde se révèle par des douleurs souvent nocturnes, habituellement calmées par la prise d'anti-inflammatoires tels que l'aspirine. Il s'agit d'une tumeur survenant dans la majorité des cas chez l'adolescent et l'adulte jeune, tumeur ayant des caractéristiques évolutives propres. Elle peut être à l'origine de lésions articulaires irréversibles lorsque proche d'une articulation. Le traitement est essentiellement chirurgical et depuis une vingtaine d'années, le traitement percutané (résection ou destruction par chauffage) a supplanté l'exérèse « en bloc » à ciel ouvert. Nous rapportons un cas rare d'ostéome ostéoïde du toit de l'acétabulum chez un jeune patient de 17 ans sans antécédents particulier et rapportant des douleurs de la hanche droite d'allure inflammatoire évoluant depuis 7 mois et sensibles a l'aspirine. La radiographie standard n'as pas objectivé de lésion visible, néanmoins le complément scannographique a mis en évidence une image lacunaire arrondie avec une calcification centrale et ostéosclérose réactionnelle évoquant un ostéome ostoïde du toit de l'acétabulum droit. Le patient a bénéficié d'une résection percutanée de la tumeur sous control scannographique. Un control clinique et radiologique après un recul de 12 mois n'a pas objectivé de récidive.

**Figure 1:**
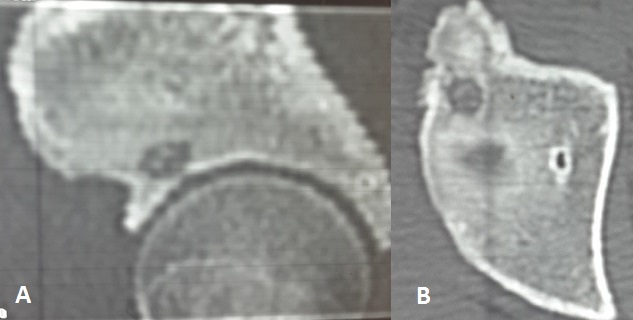
(A) TDM en coupe frontale; (B) TDM en coupe axiale montrant la lacune

